# Dual-Task Processing With Identical Stimulus and Response Sets: Assessing the Importance of Task Representation in Dual-Task Interference

**DOI:** 10.3389/fpsyg.2018.01031

**Published:** 2018-06-25

**Authors:** Eric H. Schumacher, Savannah L. Cookson, Derek M. Smith, Tiffany V. N. Nguyen, Zain Sultan, Katherine E. Reuben, Eliot Hazeltine

**Affiliations:** ^1^School of Psychology, Georgia Institute of Technology, Atlanta, GA, United States; ^2^Helen Wills Neuroscience Institute, University of California, Berkeley, Berkeley, CA, United States; ^3^Department of Psychology, University of Iowa, Iowa City, IA, United States

**Keywords:** cognitive control mechanisms, mental representation, multi-task learning, psychological refractory period, response selection

## Abstract

Limitations in our ability to produce two responses at the same time – that is, dual-task interference – are typically measured by comparing performance when two stimuli are presented and two responses are made in close temporal proximity to when a single stimulus is presented and a single response is made. While straightforward, this approach leaves open multiple possible sources for observed differences. For example, on dual-task trials, it is typically necessary to identify two stimuli nearly simultaneously, whereas on typical single-task trials, only one stimulus is presented at a time. These processes are different from selecting and producing two distinct responses and complicate the interpretation of dual- and single-task performance differences. Ideally, performance when two tasks are executed should be compared to conditions in which only a single task is executed, while holding constant all other stimuli, response, and control processing. We introduce an alternative dual-task procedure designed to approach this ideal. It holds stimulus processing constant while manipulating the number of “tasks.” Participants produced unimanual or bimanual responses to pairs of stimuli. For one set of stimuli (two-task set), the mappings were organized so an image of a face and a building were mapped to particular responses (including no response) on the left or right hands. For the other set of stimuli (one-task set), the stimuli indicated the same set of responses, but there was not a one-to-one mapping between the individual stimuli and responses. Instead, each stimulus pair had to be considered together to determine the appropriate unimanual or bimanual response. While the stimulus pairs were highly similar and the responses identical across the two conditions, performance was strikingly different. For the two-task set condition, bimanual responses were made more slowly than unimanual responses, reflecting typical dual-task interference, whereas for the one-task set, unimanual responses were made more slowly than bimanual. These findings indicate that dual-task costs occur, at least in part, because of the interfering effects of task representation rather than simply the additional stimulus, response, or other processing typically required on dual-task trials.

## Introduction

In daily life, we must often process multiple stimuli and make multiple responses in close temporal proximity, which often produces a substantial decrease in performance on one or more of the tasks performed. This so-called multi-task (or dual-task) interference has been the subject of a large number of studies over the past 50 years (for review see Pashler, 1994). Nearly all of this research involves varying the overlap between the performance of one set of stimulus-response (S–R) mappings with a different set. For example, a popular procedure for studying dual-task interference is the psychological refractory period (PRP) procedure (e.g., Welford, 1952; Pashler, 1984). This procedure involves varying the stimulus-onset asynchrony (SOA) between stimuli associated with two distinct S–R mapping sets (tasks) and measuring the decrease in task performance as SOA decreases. This decrease in performance (i.e., dual-task cost) is hypothesized to be due to processing delays caused by the task overlap. The PRP procedure has been a useful technique for identifying the locus of dual-task interference when it exists (McCann and Johnston, 1992; Schumacher et al., 1999) – in response selection processes (i.e., the mental operations that associate task-related responses to current stimuli).

Despite the fruitfulness of this and other dual-task procedures, they have some limitations for identifying *task*-*related*, versus other, sources of interference. An implicit assumption with most, if not all, dual-task procedures is that adding one or more S–R mapping sets is equivalent to adding a task. Therefore, any decrease in performance due to increased temporal overlap between the performance of the S–R mapping sets is *dual-task* interference. There is a good reason for this assumption. However, processing of the additional stimuli or responses, or other control processes may also interfere with performance regardless of the participants’ internal task representation.

Furthermore, we have shown that changing the temporal overlap between the tasks (e.g., simultaneous stimulus presentation) and the implicit and explicit priorities between them can demonstrate dual-task costs not strictly related to structural limitations in multi-tasking ability (Schumacher et al., 2001; Hazeltine et al., 2002; but see Byrne and Anderson, 2001; Anderson et al., 2005). However, simultaneous presentation of multiple-task stimuli exasperates the problem of isolating the interfering effect of task-related overlap. Thus, while the PRP procedure has effectively demonstrated that some task processes (e.g., stimulus identification) can be performed in parallel whereas others (viz., response selection) are often performed serially for distinct tasks, there is a serious limitation. Several studies (e.g., Schumacher et al., 2001; Hazeltine et al., 2002) have demonstrated that prioritizing one of the tasks and varying the SOA can produce dual-task interference that is not observed when participants perform the tasks simultaneously under other conditions. That is, the demands of the PRP procedure appear to produce interference that reflects control processes rather than structural capacity limitations. For example, Schumacher et al. (2001) showed that two tasks that could be performed without significant dual-task costs when the stimuli were presented simultaneously nonetheless produced large dual-task costs when the SOA was varied (Halvorson et al., 2013). Thus, while data from this approach likely reveal how the timing of task processes can be controlled, they may be less informative about the magnitude of interference between multiple task representations.

Here we describe a novel experimental procedure that overcomes this limitation and allows us to investigate the interference associated with performing two tasks unconfounded by the potential interference of processing multiple stimuli and responses. In this *task manipulation* procedure, participants make manual responses with both hands to the presentation of a face and building image. Some of the stimuli require responses and others do not. Participants perform two conditions. In the independent condition, the S–R mappings for the faces and places are independent of each other (i.e., the correct response for one does not depend on the other). In the relational condition, the S–R mappings include combinations of face and place stimuli (i.e., the correct responses are based on both stimuli). Because some stimuli for each condition are associated with no responses, each condition includes trials with both one (unimanual) and two responses (bimanual).

Critically, with this approach, we hold constant the number of stimuli presented on each trial and vary the number of responses. Because each condition has unimanual and bimanual response trials, it is possible to compute dual-task costs for them separately. We hypothesize that this manipulation of the S–R mapping conditions will produce a difference in the number of tasks participants think they are performing. That is, in the independent condition, participants will perform two tasks when there are two responses and one task when there is one response. Thus, reaction times (RTs) should be longer for stimuli that require two responses. In contrast, in the relational condition, participants will perform one task (which involves integrating two stimuli) no matter how many responses are produced, so RT should not depend on the number of responses. That is, the only difference between the conditions is whether participants represent the face and building stimuli as associated with separate S–R mapping sets or two tasks (viz., in the independent condition) or as part of a larger related S–R mapping set or single task (viz., in the relational condition).

The definition of “task” is often not made explicit in the literature. It may refer both to the activities required of the participant in an experiment as well as the participants’ internal representation of those activities. While there is often complete overlap between those definitions in most experiments, here we make a distinction between participants’ behavior and how they represent that behavior. Fundamentally, this is a question about how task representation affects performance. There are many theories for how information is represented and how its representation may affect behavior. In the 1980s, Norman and Shallice (1986) outlined how mental schema (complex associations between stimuli and responses) may guide behavior with and without the help of a supervisory attentional system. Kahneman et al. (1992) proposed that visual perception involved the formation of *object files*, described how attention may be allocated to task-relevant features to bind representations. Building on this idea, Hommel (1998, 2004) suggested that response selection involved the formation of *event files*, that included episodic information about both stimuli and responses. Recently, Schumacher and Hazeltine (2016), Hazeltine and Schumacher (2016) proposed the need to include another level in this representational hierarchy – namely that of a task. These *task files* include associations between stimuli and responses, contextual information, internal goals, and other relevant task information. Importantly, boundaries between task files may segregate the effects of interference in response selection (e.g., Hazeltine et al., 2011).

Because this research involves the effects of task representations – and these representations must be learned (Wilson and Niv, 2012) – we had participants practice the conditions over three experimental sessions so that we could be confident that their task representations were stable before we investigated the effect on dual-task interference. Additionally, because pilot testing showed that the relational condition was more difficult than the independent condition and we wanted to compare performance at similar levels of accuracy, the relational condition was practiced more than the independent condition.

## Materials and Methods

### Participants

Sixteen participants (age range: 18–29 years; nine female) participated in this experiment in partial fulfillment of a course requirement. This study was carried out in accordance with the recommendations of the Georgia Institute of Technology, Institutional Review Board. The protocol was approved by the Institutional Review Board. All participants gave written informed consent in accordance with the Declaration of Helsinki.

### Stimuli

Six grayscale male face images were used from the AR Face Database (Martinez and Benavente, 1998). The images included the neck, shoulders, and hair of the models. Six grayscale images of buildings (places) were also used. Three of each image type were randomly assigned to the independent and relational conditions. For the independent condition, each of the three faces were assigned to the left middle-finger response, the left index-finger response, and to no response. Place images were assigned in a similar fashion to the right middle-finger, right index-finger, and no response. For the relational condition, the other set of face stimuli were assigned to the left middle, left index, and no response conditions and the other set of places was assigned to the right middle, right index, and no response conditions. **Table 1** shows the mappings for the independent and relational conditions. The difference between the conditions was that, for the independent condition, the left-hand, right-hand, and no responses were not associated with each other, but for the relational condition, the particular left-hand, right-hand, and no responses were determined by the pair of stimuli presented.

**Table 1:** Stimulus-response mappings for the two experimental conditions.

	Independent condition			
	Left middle	Left index	Right middle	Right index
Face1–Place1	X	—	X	—
Face1–Place2	X	—	—	X
Face1–Place3	X	—	—	—
Face2–Place1	—	X	X	—
Face2–Place2	—	X	—	X
Face2–Place3	—	X	—	—
Face3–Place1	—	—	X	—
Face3–Place2	—	—	—	X
Face3–Place3	—	—	—	—
	**Relational condition**			
Face1–Place1	X	—	X	—
Face1–Place2	—	—	—	—
Face1–Place3	—	X	—	X
Face2–Place1	—	X	—	—
Face2–Place2	X	—	—	X
Face2–Place3	—	—	X	—
Face3–Place1	—	—	—	X
Face3–Place2	—	X	X	—
Face3–Place3	X	—	—	—


*The X in each column indicates the correct response given the stimulus pair presented. For the independent condition, the correct response for one stimulus type does not depend on the other. For the relational condition, the correct response is determined by the pair of stimuli presented. The X’s and dashes were presented to participants as feedback.*

### Procedure

The experiment consisted of three sessions collected on separate days within 1 week while participants lay supine in a “mock” magnetic resonance imaging scanner. After obtaining informed consent on Session 1, each session began by informing/reminding participants that they would perform two conditions. For each condition, two stimuli appeared simultaneously. A face stimulus appeared to the left of fixation and a place stimulus appeared to the right. The entire stimulus array subtended approximately 2° × 14° visual angle (vertical × horizontal). Participants responded by pressing buttons with the index and middle fingers of both hands (or made no response). In the relational condition, they were instructed to respond based on “how each pair of stimuli maps to each pair of responses. Neither stimulus alone will tell you anything about either response.” In the independent condition, they were instructed that the “left stimulus will indicate left response and right stimulus will indicate right response.” Participants were encouraged to respond to each stimulus as quickly and as accurately as possible. Participants then completed a self-paced training procedure of 26 trials where each face and place image was shown with its correct response. For this phase, each trial began with a 500 ms fixation presented in the center of the screen followed by the stimuli to the left of fixation with the correct responses for the left and right hand indicated below the stimuli. The feedback display array is shown in **Table 1**. This display remained onscreen until participants pressed a key to advance to the next trial. The training began with the relational condition.

After obtaining informed consent and initial training on Session 1, Sessions 1 and 2 were identical. Both sessions included 12 blocks: eight of the relational condition and four of the independent, randomized so that two relational blocks and one independent block occurred every three blocks (super block). After every super block, participants went through the initial self-paced training procedure again. Participants received feedback about their left- and right-hand accuracy and mean RT after every block, which remained onscreen until participants were ready to begin the next block. Participants also received feedback showing the correct mapping for 1000 ms after every incorrect trial. Session 3 was identical to Sessions 1 and 2 except participants performed six blocks of each condition and did not receive trial-level feedback after errors. The first block type was selected randomly and then alternated for the rest of the session.

Each block included 18 trials (two replications of each stimulus–response pair). To control for anticipation effects, each block also included eight catch trials where no stimuli appeared and the fixation cross remained onscreen for 2500 ms. Each experiment trial began with a fixation cross presented in the center of the screen alone for 500 ms. The stimulus pair then appeared with the fixation cross for 2000 ms. Finally, the stimuli disappeared and the fixation cross remained onscreen for a 1000 ms inter-trial interval. Participants responses were collected during the stimulus display period.

## Results

The critical test for the effect of task representation on dual-task processing is in Session 3, once participants had learned the tasks. However, to investigate how these task-representational structures change through practice, we also report the data from the first two sessions. That is, we wished to determine whether the two conditions, which involved nearly identical stimulus sets and identical responses, showed similar learning rates. We report the data from the first two sessions separately from Session 3 because they used a slightly different protocol and performance stabilized by the third session.

### Sessions 1 and 2

#### Reaction Time

**FIGURE 1:**
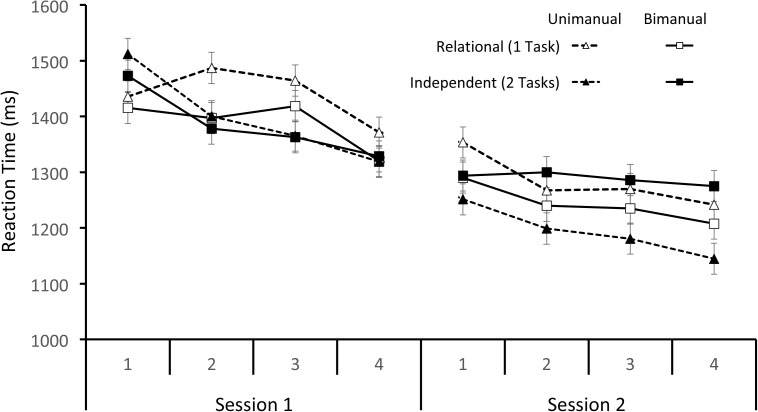
Mean RTs separated by the Mapping and Response conditions across Super Blocks for Sessions 1 and 2.

The mean RT data from Sessions 1 and 2 are shown in **Figure 1**. Trials with an incorrect response or less than 200 ms (23% total) were removed from the RT analysis. The remaining data were analyzed with a 2 × 2 × 2 × 4 within-subjects ANOVA with Condition (Relational and Independent), Response (Unimanual and Bimanual), Session (Sessions 1 and 2), and Super Block (1–4) as factors. Early in Session 1, three participants made errors on every trial in a block so their data are excluded from analysis. The data violated the assumption of sphericity so the Huynh–Feldt correction was used for all comparisons. There were significant main effects of both Session and Super Block [*F*(1,12) = 39.45, *p* < 0.001, MSE = 58,460.18, η^2^ = 0.767 and *F*(2.84,34.10) = 13.02, *p* < 0.001, MSE = 19,605.34, η^2^ = 0.520, respectively] such that participants got faster with practice. There were only two significant interactions. The interaction between Response and Session, *F*(1,12) = 5.86, *p* < 0.05, MSE = 13,584.78, η^2^ = 0.328, showed that Unimanual mean RT improved more with practice (181 ms) than Bimanual mean RT (121 ms). The interaction between Condition, Super Block, and Session, *F*(2.38,28.55) = 3.17, *p* < 0.05, MSE = 17,914.47, η^2^ = 0.209, showed that Independent condition mean RT decreased across all blocks but Relational condition mean RT did not start decreasing until Super Block 4 in Session 1.

#### Accuracy

The mean accuracy data from Sessions 1 and 2 are shown in **Table 2**. To control for possible violations of normality, the accuracy data were transformed using an arcsine transformation (arcsin(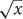)) (Sokal and Rohlf, 1995); and analyzed with a 2 × 3 × 2 × 4 within-subjects ANOVA with Condition (Relational and Independent), Response (Unimanual, Bimanual, and No Response), Session (Sessions 1 and 2), and Super Block (1–4) as factors. The data violated the assumption of sphericity so the Huynh–Feldt correction was used for all comparisons. Only significant effects will be described here. There were significant main effects of Task, Response, Super Block, and Session: *F*(1,15) = 31.21, *p* < 0.001, MSE = 0.24, η^2^ = 0.675; *F*(1.80,27.06) = 55.44, *p* < 0.001, MSE = 0.18, η^2^ = 0.787; *F*(2.82,42.36) = 88.52, *p* < 0.001, MSE = 0.06, η^2^ = 0.86; and *F*(1,15) = 114.84, *p* < 0.001, MSE = 0.27, η^2^ = 0.884, respectively. Accuracy was higher for the Independent condition than the Relational condition (84% vs. 70%). No Response, Bimanual, and Unimanual were significantly different (89%, 74%, and 68%, respectively). Accuracy increased across Super Blocks 1–4 (62%, 74%, 83%, and 89%, respectively) and across Sessions 1 and 2 (63% vs. 91%). There were also several significant interactions. The interaction between Condition and Response was significant, *F*(2,30) = 19.89, *p* < 0.001, MSE = 0.08, η^2^ = 0.57, such that accuracy for the Relational condition varied across response types more than the Independent condition. The interaction between Response and Super Block was also significant, *F*(3.17,47.49) = 4.13, *p* < 0.05, MSE = 0.07, η^2^ = 0.22, such that accuracy for the No Response condition improved more slowly than the other conditions. The interactions between Condition and Session [*F*(1,15) = 14.92, *p* < 0.05, MSE = 0.09, η^2^ = 0.50] and Response and Session [*F*(1.55, 23.25) = 9.58, *p* < 0.05, MSE = 0.12, η^2^ = 0.39] were also significant, such that accuracy for the Relational condition and the Unimanual condition improved the most from Session 1 to Session 2. The interaction between Super Block and Session [*F*(3,45) = 33.40, *p* < 0.001, MSE = 0.06, η^2^ = 0.69] was significant, such that accuracies improved more in Session 1 than Session 2. Finally, there was a significant four-way interaction between Condition, Response, Super Block, and Session, *F*(5.73,85.87) = 3.54, *p* < 0.05, MSE = 0.03, η^2^ = 0.19. Accuracies for the Unimanual and Bimanual responses in the Relational condition were quite low in Session 1 but improved so that accuracies across all conditions were similar by the end of Session 2.

**Table 2:** Mean accuracy across sessions.

	Session 1 Super block				Session 2 Super block				Session 3
Mapping condition	1	2	3	4	1	2	3	4	
Relational (1 task) unimanual	10	20	37	54	67	78	86	87	90
Relational (1 task) bimanual	19	35	62	77	81	84	85	89	91
Relational (1 task) no response	48	74	90	95	99	98	98	98	99
Independent (2 tasks) unimanual	39	64	80	89	94	94	97	96	95
Independent (2 tasks) bimanual	46	65	80	92	88	88	92	97	94
Independent (2 tasks) no response	64	89	86	95	95	100	98	97	99


### Session 3

#### Reaction Time

**FIGURE 2:**
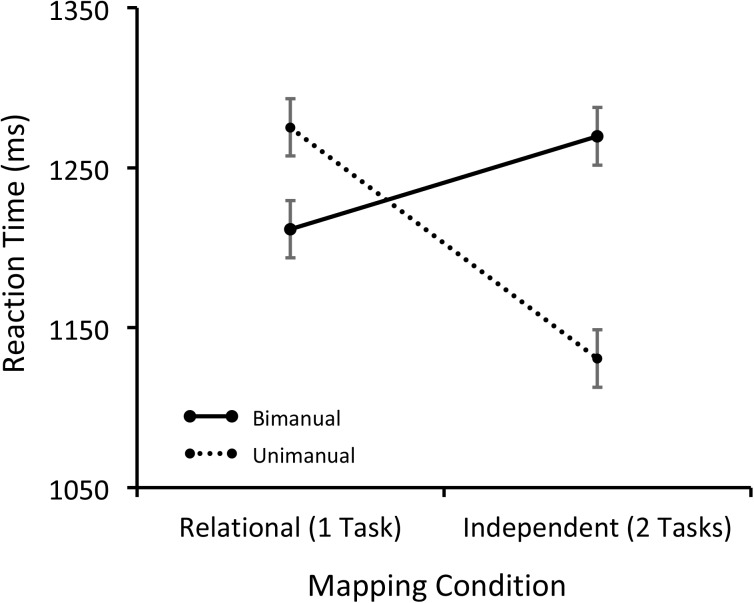
Mean RTs separated by the Mapping and Response conditions for Session 3.

Trials with incorrect responses or less than 200 ms (5% overall) were removed from the RT analysis. The mean RTs for the remaining data are shown in **Figure 2** and were analyzed with a 2 × 2 within-subjects ANOVA with Condition (Relational and Independent) and Response (Unimanual and Bimanual) as factors. Neither the effect of Condition nor Response was significant: *F*(1,15) = 2.43, *p* = 0.14, MSE = 12,332.21, *F*(1,15) = 3.48, *p* = 0.08, MSE = 6491.06, respectively. The Condition by Response interaction, however, was significant: *F*(1,15) = 32.37, *p* < 0.001, MSE = 5069.30, η^2^ = 0.683. Critically, for the Independent condition, unimanual responses were produced significantly faster than bimanual responses [*t*(15) = 4.93, *p* < 0.001], but for the Relational condition, the unimanual responses were significantly slower than bimanual ones [*t*(15) = 2.50, *p* < 0.05]. Additionally, bimanual responses did not differ between the two mapping conditions [*t*(15) = 1.56, *p* = 0.14], but unimanual responses did [*t*(15) = 5.13, *p* < 0.001].

#### Accuracy

Mean accuracies are shown in **Table 2**. To control for possible violations of normality, the accuracy data were transformed using an arcsine transformation (arcsin(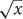)) (Sokal and Rohlf, 1995). The transformed data were analyzed with a 2 × 3 within-subjects ANOVA with Condition (Relational and Independent) and Response (Unimanual, Bimanual, and No Response) as factors. The data violated the assumption of sphericity so the Huynh–Feldt correction was used for all comparisons. The only significant effect was for Response: *F*(1.3,19.7) = 49.23, *p* < 0.001, MSE = 0.01, η^2^ = 0.766. Participants were slightly less accurate on the Relational than the Independent condition. Neither the effect of Condition nor the interaction between Condition and Response was significant: *F*(1,15) = 3.478, *p* = 0.08, MSE = 0.20, *F*(2,30) = 2.59, *p* = 0.09, MSE = 0.01, respectively.

## Discussion

The critical test of the hypothesis that task representation affects dual-task processing is tested in the data from Session 3, once participants have learned the task representations. Here despite the similarity between the stimulus and response sets used in the relational and independent conditions, participants showed distinct patterns of behavior depending on whether one or two responses were required. There was no effect of mapping or response condition on RT, but there was significant interaction between mapping condition and response (**Figure 2**). When participants considered the face and place stimuli to be part of separate task representations (the independent condition) they showed dual-task interference when making two responses, but when they considered the stimuli to be part of the same task representation (the relational condition) they did not, despite the similarity in the stimuli and responses in the two conditions. That is, performance improved when they only had to make a single response versus when they had to make two responses for the independent condition but not for the relational condition. Thus, there was a typical dual-task cost of making two manual responses to simultaneously presented stimuli versus making one manual response (e.g., Schumacher et al., 2001; Hazeltine et al., 2002), but this interference disappeared under identical stimulus and response conditions, when participants represented the stimuli and responses as part of one task. These data show, unequivocally, that the way in which people represent tasks affects how they behave.

There are several advantages to studying dual-task interference using a method like the one described here. Using simultaneous presentation of the stimuli and equal priority instructions (e.g., Schumacher et al., 2001; Hazeltine et al., 2002) may discourage strategic dual-task slowing (c.f., Meyer and Kieras, 1997a,b). However, the more novel contribution of this procedure is the way it isolates the interfering effects of task representations across the two mapping conditions. The between condition comparisons allow for the manipulation of the number of performed tasks while keeping the number of stimuli and responses constant.

Interpreting data from conventional approaches require one to assume the stimulus, response, and/or other control processing does not change across conditions. These ancillary processing assumptions are not required with the current approach. This may be particularly useful when studying the neural effects of dual-task interference where lack of control over stimulus, response, and control processing may lead to extraneous and difficult to interpret patterns of brain activity. For example, many dual-task neuroimaging studies associate prefrontal and parietal regions for dual-task processing (for a review, see Marois and Ivanoff, 2005), however others do not (e.g., Jiang et al., 2004) and Nijboer et al. (2014) suggest there is no specific region associated with dual-task processing, rather dual-task interference is due to overlap in the network of brain regions involved in task processing. This inconsistency may be caused by the poor control over the processing requirements across single- and dual-task conditions in those studies.

Although not the focus of the current research, the data obtained during training are also informative. Across Sessions 1 and 2, RTs decreased for both the relational and independent conditions. Accuracies were quite different – especially in Session 1 – between the two conditions. Accuracy was worse in the relational condition than the independent condition (despite the increased practice with this condition) through most of Sessions 1 and 2 – though accuracies were above 85% by the end of Session 2 for all conditions. This shows that the way participants represented the S–R pairs affected their ability to learn the responses. It was easier to learn the S–R pairs when they were part of separate task files than when they were part of the same one.

Despite the potential benefit of this procedure for studying dual-task interference, there are several limitations with the current research. For the relational condition, the RTs in Session 3 are closer to the bimanual independent RTs than the unimanual independent RTs (**Figure 2**). Therefore, it could be argued that both response conditions in the relational condition were affected by dual-task interference. We think this is unlikely because one would expect the RTs for the relational condition to be longer than the independent condition because it requires participants to represent a larger S–R mapping set (nine S–R pairs vs. three pairs for each task).

Another potential limitation is that the unimanual responses were significantly slower than the bimanual responses for the Relational condition on Session 3. This dual-task benefit was not predicted and it is difficult to know how to interpret it. The bimanual responses were not significantly different between the two mapping conditions so the difference between the unimanual and bimanual responses for the relational condition may be spurious. Alternatively, participants may have had a bias for making two responses in the relational condition and this may have caused additional slowing on unimanual trials. A third possibility is that the relationship between the participants’ task representation and stimulus display may produce more complex behavioral outcomes than simply the presence or absence of dual-task interference. Wickens and Carswell (1995) have proposed a proximity compatibility principle describing how factors such as the physical similarity between stimuli may facilitate or disrupt performance depending on whether participants have to integrate the stimuli (as in the relational condition) or respond to them independently. This principle is typically applied to complex displays (e.g., airplane cockpits) so more research is necessary to understand how they apply to the current procedure.

Finally, the present experiment is not able to identify the cause of the dual-task interference in the independent condition. It may be caused by bottlenecks in response selection or response production, or response grouping (Pashler, 1994). Nevertheless, the procedure described here demonstrates that performing two tasks produces interference even when the stimulus and response requirements are held constant. These data indicate that it is the requirement to maintain and select between two task sets that affect performance in dual-task situations and not only ancillary differences in stimulus and response processing. These results highlight the importance of considering task file representations when considering controlled processing requirements (c.f., Hazeltine and Schumacher, 2016; Schumacher and Hazeltine, 2016).

## Author Contributions

EH and ES conceived the research idea. DS, EH, ES, SC, and TN conducted the analyses. DS, EH, ES, KR, SC, TN, and ZS designed the experiment. EH, ES, and SC wrote the manuscript. DS, EH, ES, KR, SC, and TN edited the manuscript. DS, SC, KR, TN, and ZS collected the data.

## Conflict of Interest Statement

The authors declare that the research was conducted in the absence of any commercial or financial relationships that could be construed as a potential conflict of interest.
